# Supplementary material for: The Significance of Longitudinal Psoas Muscle Loss in Predicting the Maintenance Efficacy of Durvalumab Treatment Following Concurrent Chemoradiotherapy in Patients with Non-Small Cell Lung Cancer: A Retrospective Study
Source: Cancers (Basel). 2024 Aug 30;16(17):3037. doi: 10.3390/cancers16173037 (PMC11394210; doi:10.3390/cancers16173037)
Supplement: Supplementary file 1 [file cancers-16-03037-s001.zip › Supplementary Figure legend.docx]

**Figure S1**: **The ROC curve analysis for cut-off values of muscle loss rate**

Muscle loss rate was categorized according to the cut-off values determined by ROC analysis, and the cut-off is 6.6%.

**Figure S2**: **Comparison of the treatment effect of durvalumab between the sarcopenia and non-sarcopenia groups**

We compared progression-free survival (PFS) and overall survival (OS) between the sarcopenia and non-sarcopenia groups. (A) Kaplan–Meier curve for PFS in patients with NSCLC with and without sarcopenia prior to chemoradiotherapy. (B) Kaplan–Meier curves for OS in patients with NSCLC with and without sarcopenia prior to chemoradiotherapy. (C) Kaplan–Meier curve for PFS in patients with NSCLC with and without sarcopenia before durvalumab administration. (D) Kaplan–Meier curve for OS in patients with NSCLC with and without sarcopenia before durvalumab administration.

NSCLC: non-small cell lung cancer, PFS: progression free survival, OS: Overall survival

**Figure S3**: **Comparison of durvalumab efficacy based on longitudinal muscle loss and sarcopenia status: before chemoradiotherapy and durvalumab administration**

We categorized patients with NSCLC into four groups based on longitudinal muscle loss combined with the presence or absence of sarcopenia at two specific time points: before chemoradiotherapy and before durvalumab administration. Kaplan–Meier curves comparing durvalumab PFS for these groups, with sarcopenia and muscle loss status before chemoradiotherapy, are shown in (A). Similarly, Kaplan–Meier curves for groups categorized by sarcopenia and muscle loss status before durvalumab administration are shown in (B).

NSCLC: non-small cell lung cancer, PFS: progression free survival
