# Supplementary material for: The Significance of Longitudinal Psoas Muscle Loss in Predicting the Maintenance Efficacy of Durvalumab Treatment Following Concurrent Chemoradiotherapy in Patients with Non-Small Cell Lung Cancer: A Retrospective Study
Source: Cancers (Basel). 2024 Aug 30;16(17):3037. doi: 10.3390/cancers16173037 (PMC11394210; doi:10.3390/cancers16173037)
Supplement: Supplementary file 1 [file cancers-16-03037-s001.zip › Supplementary Figure S1.pptx]

## Slide 1
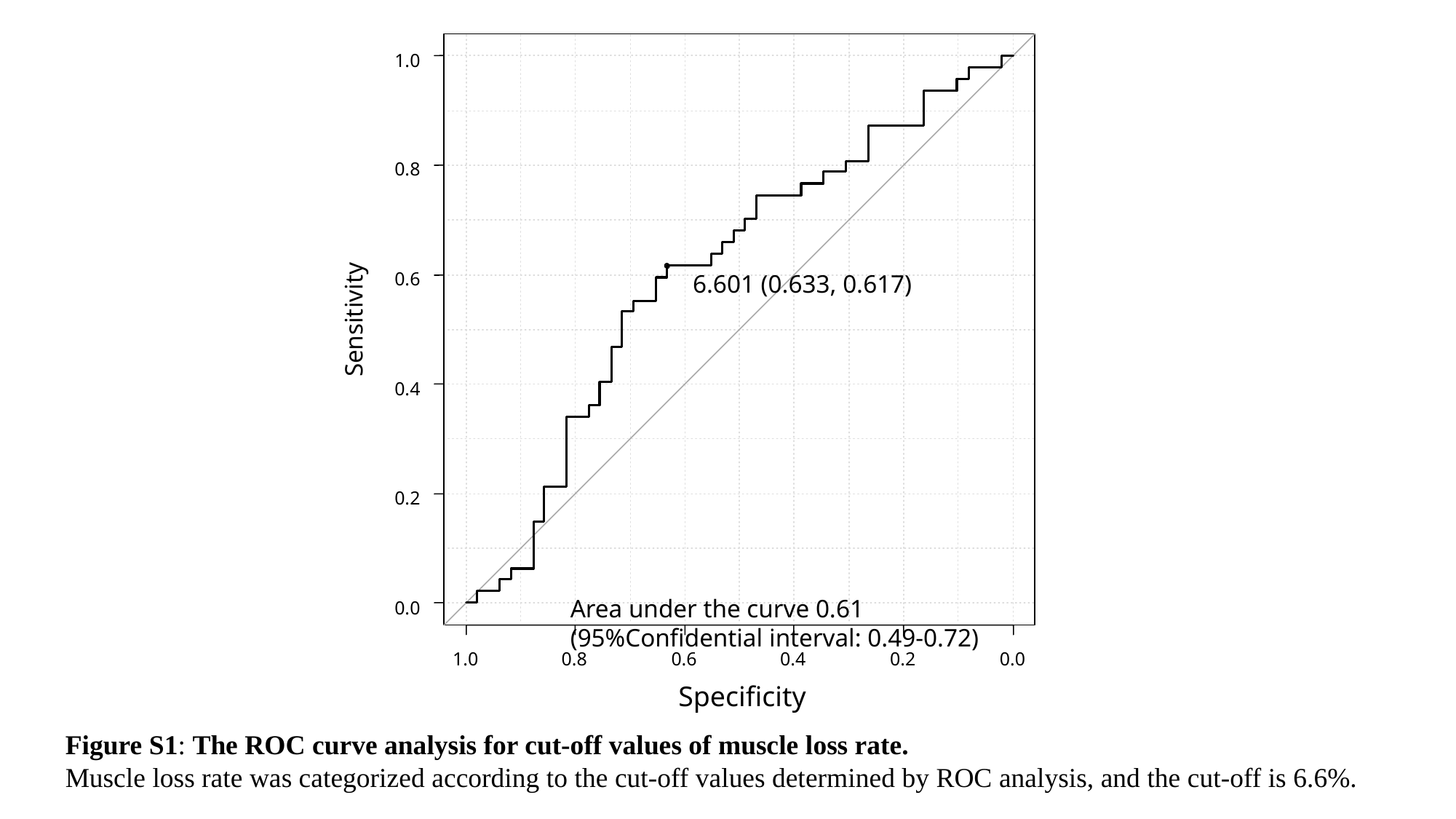

1.0
0.8
0.6
6.601 (0.633, 0.617)
Sensitivity
0.4
0.2
0.0
1.0
0.8
0.6
0.4
0.2
0.0
Specificity
Area under the curve 0.61 (95%Confidential interval: 0.49-0.72)
Figure S1: The ROC curve analysis for cut-off values of muscle loss rate.
Muscle loss rate was categorized according to the cut-off values determined by ROC analysis, and the cut-off is 6.6%.
