# Supplementary material for: The Significance of Longitudinal Psoas Muscle Loss in Predicting the Maintenance Efficacy of Durvalumab Treatment Following Concurrent Chemoradiotherapy in Patients with Non-Small Cell Lung Cancer: A Retrospective Study
Source: Cancers (Basel). 2024 Aug 30;16(17):3037. doi: 10.3390/cancers16173037 (PMC11394210; doi:10.3390/cancers16173037)
Supplement: Supplementary file 1 [file cancers-16-03037-s001.zip › Supplementary Figure S2.pptx]

## Slide 1
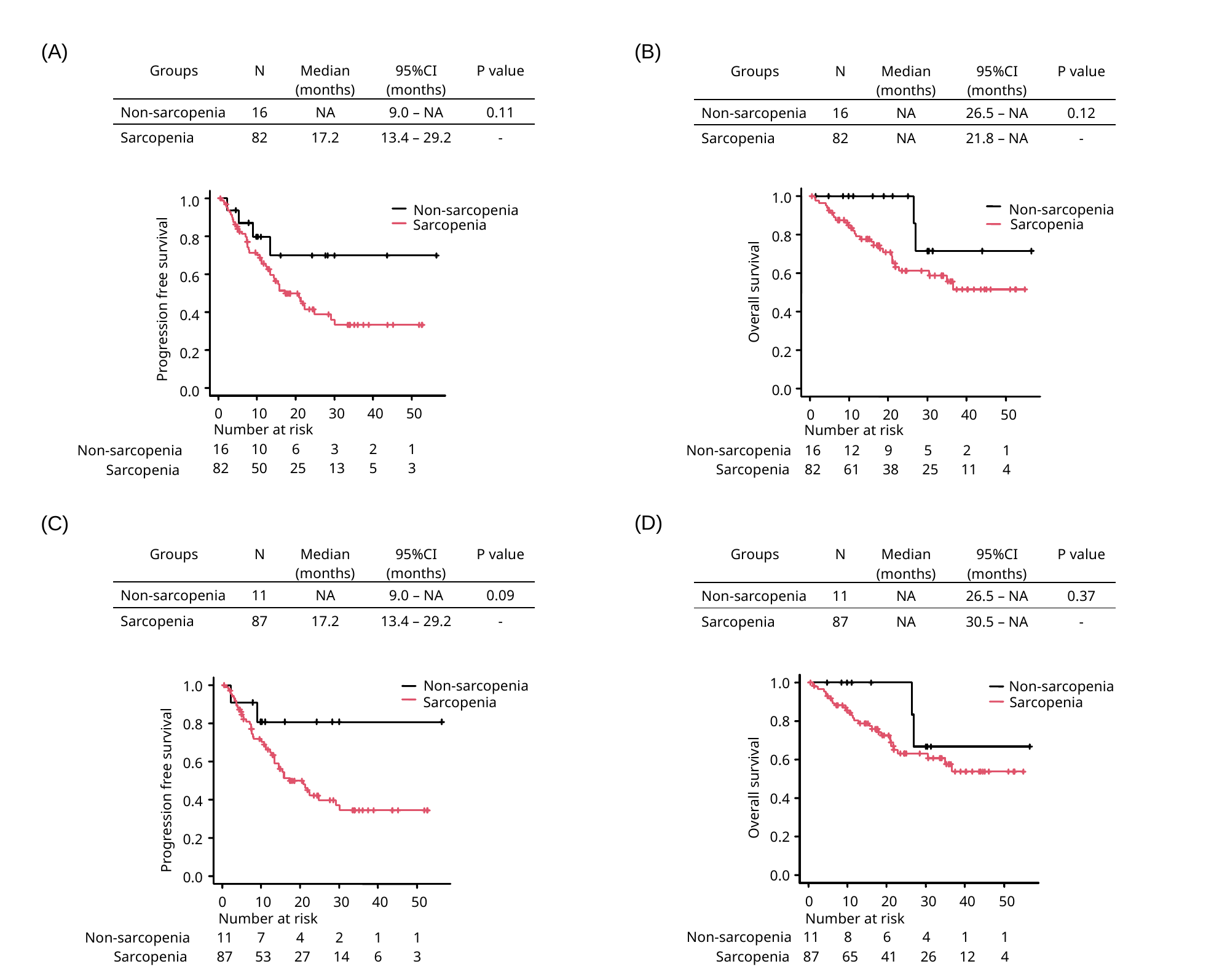

(A)
(B)
| Groups | N | Median (months) | 95%CI (months) | P value |
| --- | --- | --- | --- | --- |
| Non-sarcopenia | 16 | NA | 9.0 – NA | 0.11 |
| Sarcopenia | 82 | 17.2 | 13.4 – 29.2 | - |
| Groups | N | Median (months) | 95%CI (months) | P value |
| --- | --- | --- | --- | --- |
| Non-sarcopenia | 16 | NA | 26.5 – NA | 0.12 |
| Sarcopenia | 82 | NA | 21.8 – NA | - |
1.0
Non-sarcopenia
Sarcopenia
0.8
0.6
Overall survival
0.4
0.2
0.0
0
10
20
30
40
50
Number at risk
Non-sarcopenia
16
12
9
5
2
1
Sarcopenia
82
61
38
25
11
4
1.0
Non-sarcopenia
Sarcopenia
0.8
0.6
Progression free survival
0.4
0.2
0.0
0
10
20
30
40
50
Number at risk
16
10
6
3
2
1
Non-sarcopenia
82
50
25
13
5
3
Sarcopenia
(D)
(C)
| Groups | N | Median (months) | 95%CI (months) | P value |
| --- | --- | --- | --- | --- |
| Non-sarcopenia | 11 | NA | 9.0 – NA | 0.09 |
| Sarcopenia | 87 | 17.2 | 13.4 – 29.2 | - |
| Groups | N | Median (months) | 95%CI (months) | P value |
| --- | --- | --- | --- | --- |
| Non-sarcopenia | 11 | NA | 26.5 – NA | 0.37 |
| Sarcopenia | 87 | NA | 30.5 – NA | - |
1.0
Non-sarcopenia
Sarcopenia
0.8
0.6
Overall survival
0.4
0.2
0.0
0
10
20
30
40
50
Number at risk
Non-sarcopenia
11
8
6
4
1
1
Sarcopenia
87
65
41
26
12
4
Non-sarcopenia
1.0
Sarcopenia
0.8
0.6
Progression free survival
0.4
0.2
0.0
0
10
20
30
40
50
Number at risk
Non-sarcopenia
11
7
4
2
1
1
Sarcopenia
87
53
27
14
6
3

## Slide 2
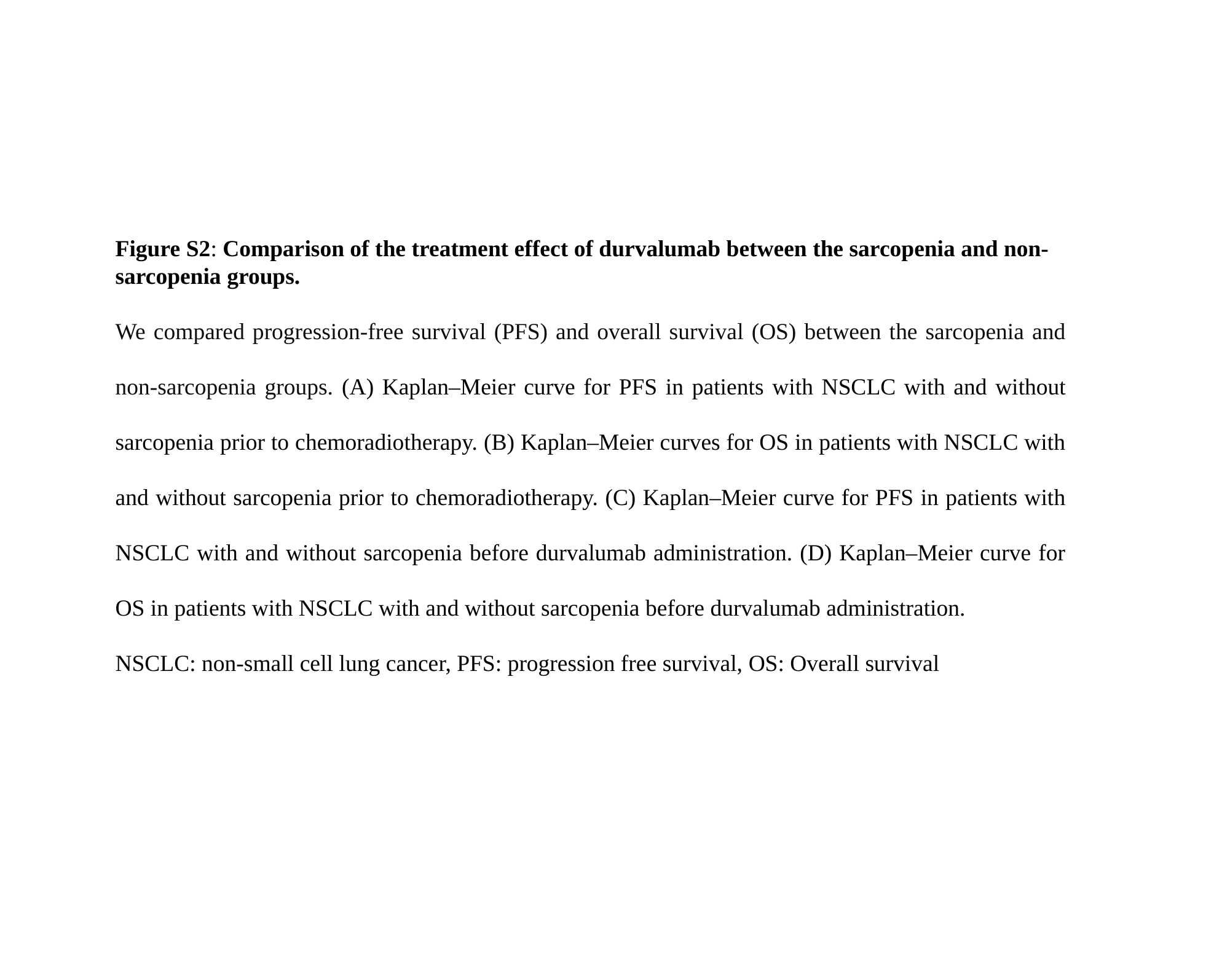

Figure S2: Comparison of the treatment effect of durvalumab between the sarcopenia and non-sarcopenia groups.
We compared progression-free survival (PFS) and overall survival (OS) between the sarcopenia and non-sarcopenia groups. (A) Kaplan–Meier curve for PFS in patients with NSCLC with and without sarcopenia prior to chemoradiotherapy. (B) Kaplan–Meier curves for OS in patients with NSCLC with and without sarcopenia prior to chemoradiotherapy. (C) Kaplan–Meier curve for PFS in patients with NSCLC with and without sarcopenia before durvalumab administration. (D) Kaplan–Meier curve for OS in patients with NSCLC with and without sarcopenia before durvalumab administration.
NSCLC: non-small cell lung cancer, PFS: progression free survival, OS: Overall survival
