# Supplementary material for: The Significance of Longitudinal Psoas Muscle Loss in Predicting the Maintenance Efficacy of Durvalumab Treatment Following Concurrent Chemoradiotherapy in Patients with Non-Small Cell Lung Cancer: A Retrospective Study
Source: Cancers (Basel). 2024 Aug 30;16(17):3037. doi: 10.3390/cancers16173037 (PMC11394210; doi:10.3390/cancers16173037)
Supplement: Supplementary file 1 [file cancers-16-03037-s001.zip › Supplementary Figure S3.pptx]

## Slide 1
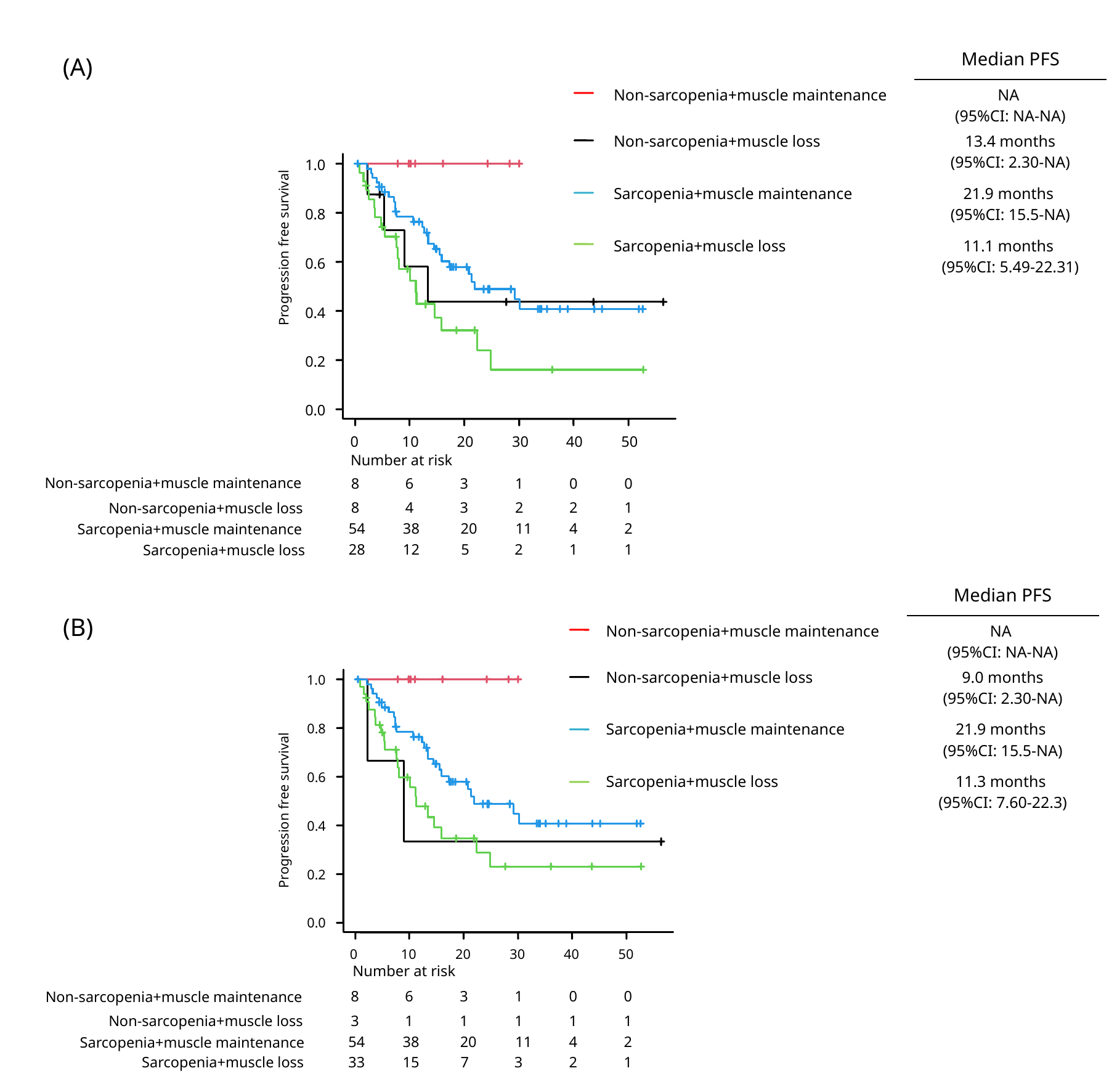

| | Median PFS |
| --- | --- |
| Non-sarcopenia+muscle maintenance | NA (95%CI: NA-NA) |
| Non-sarcopenia+muscle loss | 13.4 months (95%CI: 2.30-NA) |
| Sarcopenia+muscle maintenance | 21.9 months (95%CI: 15.5-NA) |
| Sarcopenia+muscle loss | 11.1 months (95%CI: 5.49-22.31) |
(A)
1.0
0.8
0.6
0.4
0.2
0.0
0
10
20
30
40
50
Number at risk
Non-sarcopenia+muscle maintenance
8
6
3
1
0
0
8
4
3
2
2
1
Non-sarcopenia+muscle loss
54
38
20
11
4
2
Sarcopenia+muscle maintenance
28
12
5
2
1
1
Sarcopenia+muscle loss
Progression free survival
| | Median PFS |
| --- | --- |
| Non-sarcopenia+muscle maintenance | NA (95%CI: NA-NA) |
| Non-sarcopenia+muscle loss | 9.0 months (95%CI: 2.30-NA) |
| Sarcopenia+muscle maintenance | 21.9 months (95%CI: 15.5-NA) |
| Sarcopenia+muscle loss | 11.3 months (95%CI: 7.60-22.3) |
(B)
1.0
0.8
0.6
0.4
0.2
0.0
0
10
20
30
40
50
Number at risk
Progression free survival
8
6
3
1
0
0
Non-sarcopenia+muscle maintenance
Non-sarcopenia+muscle loss
3
1
1
1
1
1
54
38
20
11
4
2
Sarcopenia+muscle maintenance
Sarcopenia+muscle loss
33
15
7
3
2
1

## Slide 2
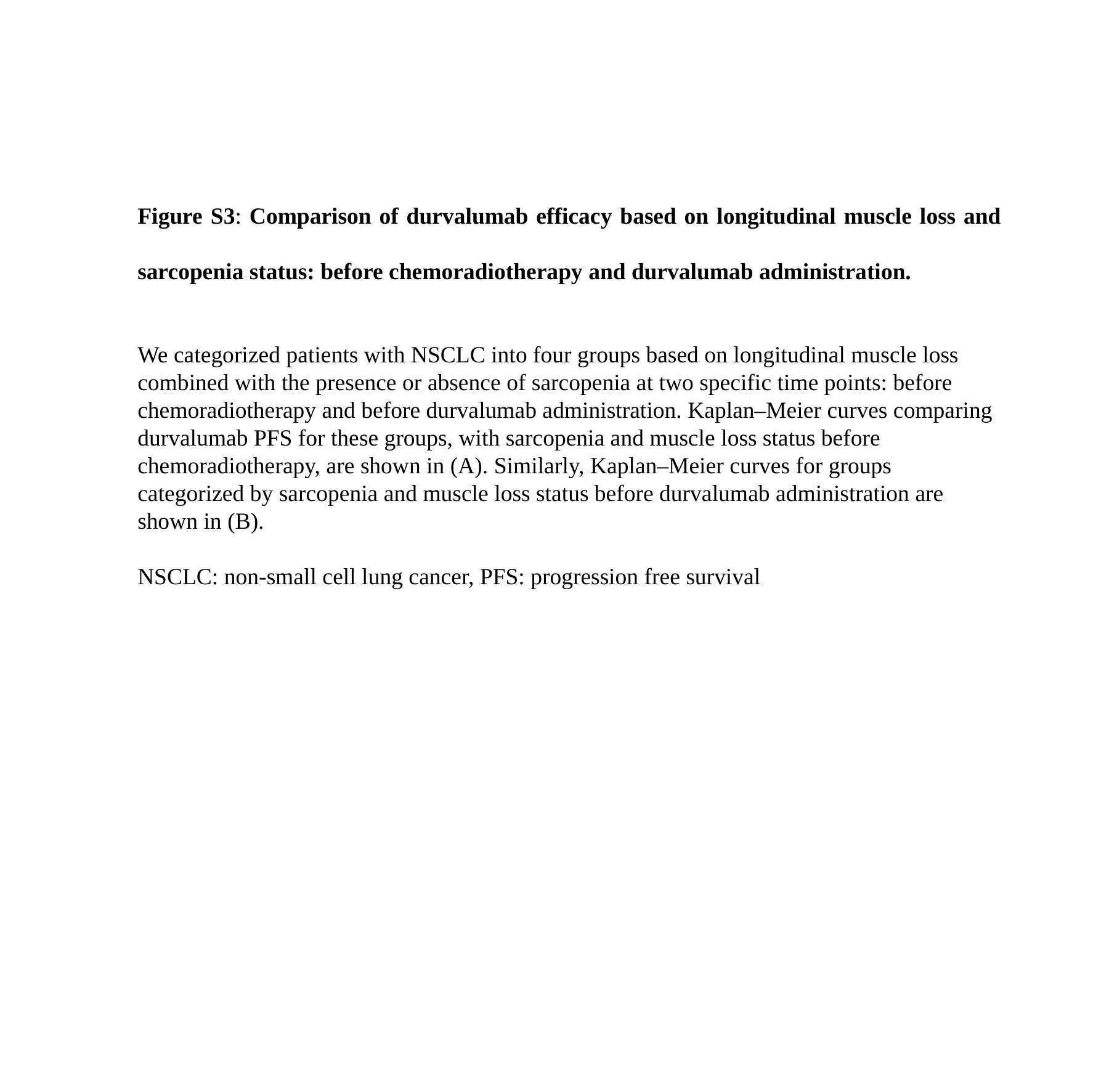

Figure S3: Comparison of durvalumab efficacy based on longitudinal muscle loss and sarcopenia status: before chemoradiotherapy and durvalumab administration.
We categorized patients with NSCLC into four groups based on longitudinal muscle loss combined with the presence or absence of sarcopenia at two specific time points: before chemoradiotherapy and before durvalumab administration. Kaplan–Meier curves comparing durvalumab PFS for these groups, with sarcopenia and muscle loss status before chemoradiotherapy, are shown in (A). Similarly, Kaplan–Meier curves for groups categorized by sarcopenia and muscle loss status before durvalumab administration are shown in (B).
NSCLC: non-small cell lung cancer, PFS: progression free survival
